# Orally administered liposomal formulations for colon targeted drug delivery

**DOI:** 10.3389/fphar.2014.00138

**Published:** 2014-06-10

**Authors:** Susan Hua

**Affiliations:** School of Biomedical Sciences and Pharmacy, The University of NewcastleCallaghan, NSW, Australia

**Keywords:** colon targeted drug delivery, nanoparticles, liposomes, oral administration, inflammatory bowel disease, inflammation

Colon targeted drug delivery is an active area of research for diseases affecting the colon, as it shows promise in improving the efficacy of therapeutics and reducing systemic toxicity. Improved oral drug delivery design has unequivocally improved the bioavailability of drugs to the colon. The oral route of administration is the most common method of drug delivery. It is the preferred route of administration because of increased patient convenience and reduced invasiveness. Rectal formulations may seem ideal for colon targeted drug delivery; however in addition to the issues surrounding patient administration, the efficacy of these formulations is limited to conditions affecting the distal colon and rectum. They are not effective for the treatment of more widespread inflammation of the colon, which occurs in conditions such as Inflammatory Bowel Disease (IBD).

Oral formulations can be designed to achieve either a local or systemic delivery of therapeutics. Local treatment requires that the drug will be delivered to the site of action within the gastrointestinal (GI) tract to have a localized effect, but will not be absorbed or only poorly absorbed. Systemic delivery for orally administered drugs requires systemic absorption occurring within the intestines prior to distribution of drug around the body. For conditions involving localized inflammation to the tissues of the colon, the ideal formulation would be one that (i) is administered orally, (ii) is able to reach the colon, (iii) delivers high concentrations of drug at the site of inflammation, and (iv) is not systemically absorbed.

Current formulations have limited effect on specificity of targeting to diseased colon tissue vs. healthy colon tissue. In addition, despite coverage to the surface of the colon (including diseased tissue), there is no guarantee that the drug is effectively taken up into the tissue and cells at the site of inflammation. Therefore, the use of nanotechnology in formulation design has been investigated as a way to further improve the efficacy of therapeutics by allowing specific targeting and uptake into inflamed tissue within the colon (Jani et al., 1989, 1990).

Nanoformulations have been developed for the targeted treatment of a number of pathological conditions, including IBD, cancers, and infections (Vingerhoeds et al., 1994; Singh, 1999; Maruyama, 2002). For the purpose of GI tract targeting, liposomes can be manipulated by the inclusion of polymer coatings on the liposomal surface. These coatings allow oral liposomal formulations to resist degradation in the hostile environment of the GI tract, which include bile salts and enzymes (e.g., pancreatic lipases) that would normally dissolve the lipid bilayer (Iwanaga et al., 1999; Takeuchi et al., 2003; Karn et al., 2011). This article will briefly discuss strategies which have been investigated for targeting drug-encapsulated liposomes to the colon.

## Natural polymers—chitosan and pectin

Chitosan and pectin are natural polymers that are considered non-toxic, biodegradable, and mucoadhesive. Adhesion to the mucosa is an advantage for GI tract targeting as it promotes the direct contact of the liposomes to the mucosal surface for cellular uptake and drug release, and reduces the clearance of liposomes when intestinal motility is increased, which occurs in conditions such as IBD (Thirawong et al., 2008a,b; Mady et al., 2009; Nguyen et al., 2011; Han et al., 2012). The mucoadhesive property of natural polymers is thought to be due to the electrostatic interaction between the cationic polymer, and the anionic sulfonic and sialic acid residues within the mucus matrix (Han et al., 2012).

Chitosan-coated liposome formulations have shown increased stability in simulated gastric and intestinal fluids (Han et al., 2012), as well as improved drug uptake in colon tissue *ex vivo* in comparison to uncoated liposomes (Takeuchi et al., 2003; Gradauer et al., 2012, 2013; Han et al., 2012; Manconi et al., 2013). Thirawong et al. (2008a,b) evaluated the mucoadhesion and uptake of orally-administered pectin-coated liposomes *in vivo* and was able to demonstrate increase residence of these nanoparticles in the GIT mucosa, with very little colloidal aggregation (Thirawong et al., 2008b). However, the majority of the formulation was found to accumulate in the small intestine, with little uptake in the colon (Thirawong et al., 2008b). The use of colon-specific enteric coating such as Eudragit® may be used to overcome this issue.

## Synthetic polymers—Eudragits^®^

Eudragit® is a synthetic copolymer coating that combines both the mucoadhesive and pH-dependent release strategies, to enhance colon targeted drug delivery via the oral route of administration. Eudragits® are methacrylic co-polymers with varying side group compositions that alter the pH at which they are soluble (Barea et al., 2012). They are widely used in oral pharmaceutics as enteric coatings of tablets and capsules (Khan et al., 1999, 2000; Barea et al., 2012). Eudragit®-coated liposomal and microsphere formulations have demonstrated favorable pH-dependent release characteristics *in vitro* (Xing et al., 2003; Haznedar and Dortunc, 2004; Barea et al., 2010). For example, Barea et al. (2010) reported a significant reduction in drug release from Eudragit®-coated liposomes at pH 1.4 (stomach) and pH 6.3 (small intestine), and significant drug release at pH 7.8 (ileocaecal region) (Barea et al., 2010). The study also reported degradation of the Eudragit®-coating by bile salts *in vitro*, which would adversely affect its efficacy *in vivo* by causing premature degradation of the liposomes and release of the drug in the duodenum. Karn et al. (2011) demonstrated using freshly extracted pig intestine tissue that liposomes coated with Eudragit® have superior mucoadhesion characteristics, in comparison to other common polymers such as chitosan and carbopol (Karn et al., 2011). These studies show that the use of Eudragit® as a coating over nanoparticles would be able to target the colon and provide effective drug release, however further formulation design is required to address the vulnerability of this coating to bile salts.

## Active targeting—immunoliposomes

The use of polymers to coat liposomal formulations has enhanced drug delivery to the colon following oral administration, via pH-dependent release and mucoadhesive characteristics. However, these formulations have limited effect on specificity of targeting to diseased colon tissue vs. healthy colon tissue. Active targeting approaches with the coupling of ligands to the surface of nanoparticles (e.g., liposomes) would allow more specific targeting to regions within the colon, by exploiting disease-induced cellular changes in cell-surface receptors and proteins. The coupling of antibodies, particularly monoclonals, to create immunoliposomes represents one of the more versatile ligands that can be affixed to liposome surfaces. In particular, it has been demonstrated that specific delivery of drugs to the target cells is far more efficient with immunoliposomes than with liposomes lacking a target antibody ligand (Vingerhoeds et al., 1994; Willis and Forssen, 1998; Bendas, 2001; Maruyama, 2002). The majority of the work in the field of immunoliposome-based formulations has been studied using the parenteral route of administration (e.g., intravenous, subcutaneous, intraperitoneal injection) (Vingerhoeds et al., 1994; Willis and Forssen, 1998; Bendas, 2001; Maruyama, 2002).

Oral administration of immunoliposomes has its challenges, as the antibodies are prone to degradation by the stomach acid as well as by GI tract enzymes. In addition, the liposomal bilayer is digested by the bile salts and enzymes if not protected by an additional coating. Much of the research on targeted drug delivery to the GI tract has centered on delivery of chemotherapeutics to intestinal cancers, via liposomes conjugated to antibodies raised against receptors which are over-expressed in both tumors and tumor-associated tissues during carcinogenesis (Koning et al., 1999, 2002; Mizoue et al., 2002; Guin et al., 2011; Wicki et al., 2012). These studies are usually limited to *in vitro* or *ex vivo* cell association experiments, and have shown positive results in specificity of cell targeting and uptake. Immunoliposomes also have inherent mucopenetrative properties, which further support its use in targeting inflamed intestinal mucosal tissue (Saltzman et al., 1994; Shen et al., 2006). For example, Harel et al. (2011) demonstrated a 4-fold increase in transferrin receptor (TfR)-targeted immunoliposome uptake in freshly excised intestinal mucosal tissue from a rodent induced-colitis model of IBD in comparison to uninflamed excised rat tissue (Harel et al., 2011). These results demonstrate that the use of immunoliposomes is promising for targeting specifically inflamed regions within the colon, which occurs in conditions such as IBD.

Intercellular adhesion molecule-1 (ICAM-1) has been shown to be upregulated in inflammatory regions of the colon, which occurs in conditions causing colitis and in IBD (Binion et al., 1997, 1998; Sans et al., 1999; Danese et al., 2005). ICAM-1 is expressed more prominently on the surface of inflamed intestinal mucosal tissues and microvasculature (Salmi et al., 1994; Bernstein et al., 1996). In Crohn's Disease, ICAM-1 is also expressed in deeper tissues such as the submucosal and muscle layers, which promote transmural invasion of leukocytes (Hogaboam et al., 1996). ICAM-1 targeted nanoparticles for oral drug delivery has recently been investigated by Mane and Muro (2012). This study evaluated the biodistribution and uptake of empty anti-ICAM-1 antibody-coated nanocarriers composed of the polymer, polystyrene, in the GI tract following gastric gavage in healthy mice. As expected, approximately 60% of the antibody dose administered (1.1 mg antibody per kg) was degraded, which was attributed mainly to GI tract enzymes. The nanocarriers were deposited mainly in the stomach and duodenum, which suggest more upper GIT targeting. Using transmission electron microscopy and energy dispersive X-ray spectroscopy, they were also able to demonstrate internalization of the radiolabelled nanocarriers within duodenal tissue via ICAM-1 (Mane and Muro, 2012). This work shows the importance of ICAM-1 in the GIT, and the potential of ICAM-1 targeted nanocarriers in the delivery of therapeutic agents for the treatment of pathologies of the GIT.

## Future advances in orally administered liposomal formulations for colon targeted drug delivery

Specific drug delivery to the colon is highly desirable for local treatment of a variety of bowel diseases such as ulcerative colitis, Crohn's disease, infections and colorectal cancer. Contrary to most therapeutic regimens utilizing oral administration, systemic absorption is an undesirable delivery feature for these drugs. Disease localization dictates the need for maximal intestinal tissue drug exposure while systemic delivery should be minimized to avoid unwanted side effects. Delayed or controlled release dosage forms have proven beneficial to negate upper intestinal absorption and release the drug at the sites of disease. The potential to increase drug residence time in regions of diseased tissue would serve to further optimize this therapy. The use of nanoparticulate, mucoadhesive, and active targeting dosage forms are promising strategies to target and adhere to diseased bowel tissue, and could provide sustained exposure of a therapeutic agent to sites of pathology and improve its therapeutic effect.

The use of liposomes has been shown to selectively target inflamed tissue, with the disruption of the intestinal barrier function at the site of inflammation allowing accumulation of particulate delivery carriers. Liposomes can also be modified to enhance binding and cellular uptake to diseased tissue by the use of cationic lipids or the attachment of targeting ligands (e.g., antibodies, peptides). Future studies will need to assess whether there is a significant difference in specificity in binding and accumulation of targeted liposomes vs. charged non-targeted liposomes. In addition, a combination of measures will need to be investigated to protect the liposomes and targeting ligands from degradation in the GI tract, including from bile salts, GI enzymes and stomach acid. For example, the use of polymer coatings or encapsulating freeze dried liposomes in capsules coated with pH-dependent release polymer may be considered (Gupta et al., 2013). Additional strategies will need to address potential bile salts degradation of the liposomal and polymer coatings. Finally, further *in vivo* studies are warranted to evaluate the colon targeted oral formulations in animal models of colonic inflammation. Formulating an oral delivery system that can effectively targeted diseased tissue within the colon has the potential to be modified to encapsulate a multitude of drugs, novel compounds, or imaging agents for the treatment or management of a number of pathologies affecting the colon.

### Conflict of interest statement

The author declares that the research was conducted in the absence of any commercial or financial relationships that could be construed as a potential conflict of interest.
